# Vaccination Confidence and Parental Refusal/Delay of Early Childhood Vaccines

**DOI:** 10.1371/journal.pone.0159087

**Published:** 2016-07-08

**Authors:** Melissa B. Gilkey, Annie-Laurie McRee, Brooke E. Magnus, Paul L. Reiter, Amanda F. Dempsey, Noel T. Brewer

**Affiliations:** 1 Department of Population Medicine, Harvard Medical School & Harvard Pilgrim Health Care Institute, Boston, Massachusetts, United States of America; 2 Department of Pediatrics, University of Minnesota, Minneapolis, Minnesota, United States of America; 3 Department of Psychology, University of North Carolina, Chapel Hill, North Carolina, United States of America; 4 Division of Cancer Prevention and Control, The Ohio State University, Columbus, Ohio, United States of America; 5 Adult and Child Centered Outcomes Research and Dissemination Science (ACCORDS) Program, University of Colorado Denver, Aurora, Colorado, United States of America; 6 Department of Health Behavior and Lineberger Comprehensive Cancer Center, University of North Carolina, Chapel Hill, North Carolina, United States of America; University College Cork, IRELAND

## Abstract

**Objective:**

To support efforts to address parental hesitancy towards early childhood vaccination, we sought to validate the Vaccination Confidence Scale using data from a large, population-based sample of U.S. parents.

**Methods:**

We used weighted data from 9,354 parents who completed the 2011 National Immunization Survey. Parents reported on the immunization history of a 19- to 35-month-old child in their households. Healthcare providers then verified children’s vaccination status for vaccines including measles, mumps, and rubella (MMR), varicella, and seasonal flu. We used separate multivariable logistic regression models to assess associations between parents’ mean scores on the 8-item Vaccination Confidence Scale and vaccine refusal, vaccine delay, and vaccination status.

**Results:**

A substantial minority of parents reported a history of vaccine refusal (15%) or delay (27%). Vaccination confidence was negatively associated with refusal of any vaccine (odds ratio [OR] = 0.58, 95% confidence interval [CI], 0.54–0.63) as well as refusal of MMR, varicella, and flu vaccines specifically. Negative associations between vaccination confidence and measures of vaccine delay were more moderate, including delay of any vaccine (OR = 0.81, 95% CI, 0.76–0.86). Vaccination confidence was positively associated with having received vaccines, including MMR (OR = 1.53, 95% CI, 1.40–1.68), varicella (OR = 1.54, 95% CI, 1.42–1.66), and flu vaccines (OR = 1.32, 95% CI, 1.23–1.42).

**Conclusions:**

Vaccination confidence was consistently associated with early childhood vaccination behavior across multiple vaccine types. Our findings support expanding the application of the Vaccination Confidence Scale to measure vaccination beliefs among parents of young children.

## Introduction

From January 2014 to June 2015, the Centers for Disease Control and Prevention documented over 800 cases of measles in the United States, including a cluster of 117 cases linked to a California amusement park [1]. The highly publicized “Disneyland outbreak” brought national attention to the rising incidence of measles specifically as well as to the issue of parents’ hesitancy to vaccinate their children against infectious diseases more generally [2]. Public health research to date largely supports assertions made in the popular press that parents’ confidence in routine vaccination has eroded in recent years, leading to vaccine refusal and delay [3–6]. Evidence of this trend comes indirectly from several sources, including studies that show that parents are more often requesting exemptions to school entry requirements for vaccination and that providers perceive parental vaccine refusal as being more common than in the past [3–6]. However, neither vaccine refusal and delay, nor the underlying concerns that motivate these behaviors, are routinely assessed in the National Immunization Survey or other ongoing, large-scale surveillance efforts. For this reason, our ability to more directly quantify changes in the prevalence of vaccine hesitancy is limited.

Public health experts, including the National Vaccine Advisory Committee (NVAC), have highlighted the need to address this gap through additional research on vaccine refusal and delay [7]. In a recent report, the NVAC Vaccine Confidence Working Group placed special emphasis on developing valid and reliable measures to assess parental beliefs and other determinants of forgone vaccination [7]. Toward this end, we developed the Vaccination Confidence Scale, an 8-item, 3-factor scale that measures beliefs related to the perceived benefits of vaccination, the perceived harms of vaccination, and trust in vaccine providers [8]. We originally created our scale to assess parental beliefs related to adolescent vaccination, and in a prior validation study, we found that parents’ mean Vaccination Confidence Scale scores were consistently associated with vaccine refusal and vaccination status for vaccines in the adolescent platform, including meningococcal and human papillomavirus (HPV) vaccines [9]. This success raises the possibility that a modified version of our scale could be useful for assessing vaccination beliefs related to early childhood vaccines, including measles, mumps, and rubella (MMR) vaccine.

To investigate this question, we conducted a second validation study of the Vaccination Confidence Scale. Using data from a nationally-representative sample of parents, we sought to assess associations between vaccination confidence and vaccine refusal, vaccine delay, and vaccination status for vaccines administered in early childhood. By broadening our scale’s application to include early childhood vaccination, this study aims to support ongoing efforts to understand and increase the public’s vaccination confidence so as to reduce vaccine refusal and delay across the life course.

## Methods

### Participants and Data Source

Data came from the 2011 National Immunization Survey (NIS). As described previously, the NIS is a population-based survey conducted annually by the Centers for Disease Control and Prevention with the primary goal of assessing children’s vaccination status; in 2011, the survey also included questions to assess parents’ vaccination beliefs [10]. In the first phase of data collection, a probability sample of parents and guardians participated in a landline or cellular telephone survey; the purpose of this survey was to assess the immunization history of a randomly selected 19- to 35-month-old child in the household. (To facilitate reporting, we refer to respondents hereafter as “parents.”) For those parents who gave consent, a second phase of data collection involved a mail-based survey sent to children’s vaccine providers; the purpose of this survey was to assess children’s vaccination status using medical records.

The response rate for the 2011 NIS household survey was 62% for the landline telephone sample and 25% for the cellular telephone sample [11]. Across the samples, providers returned adequate data for 72% of all respondents. We drew our analytic sample from 12,580 parents with adequate provider-confirmed vaccination data who completed the “Parental Concerns Module,” a special set of questions included in the 2011 NIS. We excluded parents who had missing data on variables related to vaccination beliefs or behaviors (n = 2,461) or who completed the survey in a language other than English (n = 765). Our final analytic sample consisted of the remaining 9,354 parents.

The National Center for Health Statistics (NCHS) Research Ethics Review Board approved data collection for the 2011 NIS. Analysis of de-identified data from the survey is exempt from the federal regulations for the protection of human research participants. We accessed data from the Parental Concerns Module through the NCHS Research Data Center because these restricted variables are not included in the public-use dataset. Analysis of restricted data through the NCHS Research Data Center was approved by the NCHS Ethics Review Board. The University of North Carolina Institutional Review Board determined that this study was exempt from further review.

### Measures

**Fig 1:**
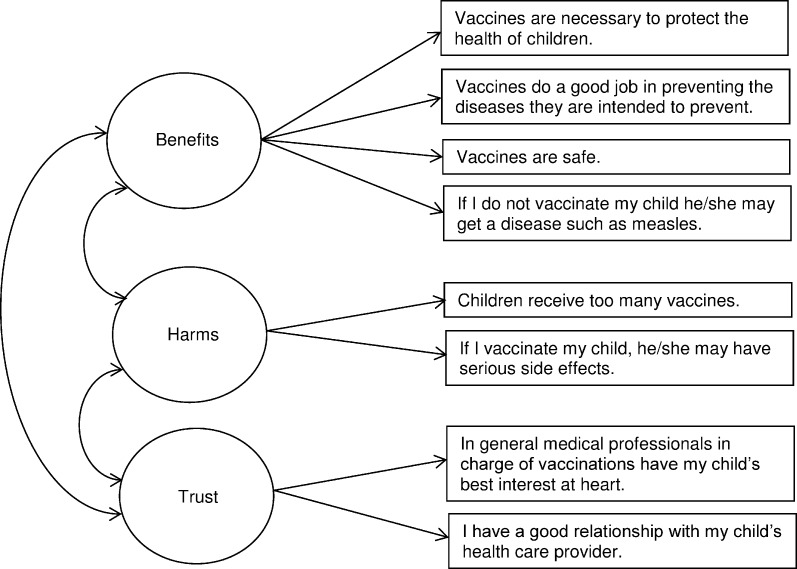
The Vaccination Confidence Scale as modified for early childhood vaccination.

The 2011 NIS Parental Concerns Module assessed parents’ vaccination beliefs with survey items that used an 11-point response scale ranging from 0 (“strongly disagree”) to 10 (“strongly agree”). The module included items corresponding to the 8 items from the 2010 NIS-Teen that we used to develop the Vaccination Confidence Scale in a prior study, with the difference being that NIS-Teen items referred to “teenagers” while NIS items referred to “children” [8]. The resulting scale consisted of factors assessing perceived benefits of adolescent vaccination (i.e., “Benefits,” α = 0.78), perceived harms (“Harms,” α = 0.56), and trust in healthcare providers (“Trust,” α = 0.55) (Fig 1). To assess overall confidence, we calculated mean scale scores for each parent by averaging responses for all 8 items, after reverse coding negative beliefs in the Harms factor. The resulting scores had a possible range of 0 to 10 with higher scores indicating more positive beliefs about vaccination. We next calculated mean scores for the Benefits, Harms (without reverse coding), and Trust factors by averaging the item responses within each factor.

The Parental Concerns Module also assessed parental refusal and delay of early childhood vaccines. The survey assessed history of any vaccine refusal with a single item: “Has there ever been a time when you refused or decided not to get a vaccination for [CHILD NAME]?” For those reporting any refusal, separate items with yes/no response options assessed whether parents had refused specific vaccines, including measles/measles-mumps-rubella/MMR; chicken pox/varicella; and seasonal influenza/flu shot/flu nasal spray/FluMist. The survey also assessed history of any vaccine delay: “Has there ever been a time when you delayed or put off getting a vaccination for [CHILD NAME]?” For those reporting any delay, parents reported which vaccines they had delayed as for vaccine refusal. Items on refusal and delay used yes/no response options.

The 2011 NIS household survey assessed demographic characteristics including the child’s age, sex, and race/ethnicity (Table 1). NCHS analysts used demographic data to determine children’s eligibility for the Vaccines for Children (VFC) program, which provides free vaccines for children whose families have limited ability to pay [12]. Survey respondents indicated their relationship to the child, the age and educational attainment of the child’s mother, and the annual income and geographic location of the household.

**Table 1:** Sample characteristics (*n* = 9,354).

	*n*	(%)
Child characteristics		
Age (montds)		
19–23	2,701	(30)
24–29	2,945	(35)
30–35	3,708	(36)
Sex		
Male	4,812	(53)
Female	4,542	(47)
Race/ethnicity		
Non-Hispanic white	6,344	(61)
Non-Hispanic black	1,002	(14)
Hispanic	1,004	(15)
Other	1,004	(10)
Vaccines for Children eligibility		
Yes	3,109	(45)
No	6,194	(54)
Not reported	51	(<1)
Parent characteristics		
Relationship to child		
Mother/female guardian	7,339	(78)
Father/male guardian	1,522	(17)
Other	493	(5)
Mother’s age		
≤ 19 years	139	(3)
20–29 years	2,676	(41)
≥ 30 years	6,539	(57)
Mother’s education		
12 years or less	2,073	(39)
Some college, no degree	2,508	(24)
College degree or more	4,773	(38)
Household characteristics		
Region		
Northeast	1,767	(18)
Midwest	2,212	(24)
South	3,419	(39)
West	1,956	(19)
Annual income^a^		
Below poverty level	1,631	(29)
Above poverty level, ≤$75,000	3,420	(37)
>$75,000	4,056	(31)
Not reported	247	(3)

*Note*. Table shows raw frequencies and weighted percentages. Percentages may not total 100% due to rounding.

^a^ Poverty level based on 2010 U.S. Census poverty threshold.

The 2011 NIS provider survey assessed the child’s vaccination status for vaccines in the routine immunization schedule. Providers used medical records to indicate the dates on which the child received vaccine doses, and NCHS analysts then determined whether the child was up-to-date by age 36 months for vaccinations including ≥1 dose of MMR, ≥1 dose of varicella, and ≥1 dose of seasonal flu vaccines [10,11]. The NCHS also reported whether children were up-to-date for the 4:3:1:-:3:1:4 combined series, which included ≥4 doses of tetanus-containing vaccine, ≥3 doses of poliovirus vaccine, ≥1 dose of measles-containing vaccine, ≥3 doses of hepatitis B, ≥1 dose of varicella vaccine, and ≥4 doses of pneumococcal conjugate vaccine (PCV). As indicated by the dash (-), this series excluded *Haemophilus* influenzae type b (Hib) due to a shortage of that vaccine during the study period.

### Statistical Analyses

To investigate the relationship between overall vaccination confidence and vaccination behavior, we used separate logistic regression models to assess the association between mean Vaccination Confidence Scale scores and vaccine refusal and delay. We examined these outcomes for any vaccine, as well as for MMR, varicella, and flu vaccines specifically. We also used logistic regression to assess the association between mean scale scores and vaccination status for the combined series, as well as for MMR, varicella, and flu vaccines separately. To explore the relative influence of each scale factor, we re-ran logistic regression models using all three factors, instead of a single overall scale score. Models controlled for three demographic factors that prior research indicates are associated with vaccine refusal or delay: child’s race/ethnicity, mother’s educational attainment, and annual household income [13–14].

Our analyses used survey weights developed by NCHS analysts to obtain nationally representative estimates. We report raw frequencies and weighted means, percentages, and odds ratios. Conducted in SAS 9.3 (Cary, NC), all statistical tests were 2-tailed with a critical alpha of 0.05.

## Results

### Sample Characteristics

Parents reported on roughly equal numbers of male (53%) and female (47%) children (Table 1). Most children were non-Hispanic white (61%), non-Hispanic black (14%), or Hispanic (15%). Almost half were eligible for the Vaccines for Children program (45%). About three-quarters of parents were mothers or female guardians (78%). A substantial minority of parents had a high school degree or less education (39%) or lived below the poverty threshold (29%).

### Vaccination Confidence

Parents reported high levels of vaccination confidence. The mean score for the full, 8-item scale was 8.30 (standard error [SE] = 0.03). Factor score means were 8.72 (SE = 0.03) for Benefits, 3.58 (SE = 0.05) for Harms (without reverse coding), and 9.34 (SE = 0.03) for Trust.

### Scale Validation

#### Vaccine refusal

Fifteen percent of parents reported having refused any vaccine for their child, with refusal of specific vaccines being 3% for MMR, 3% for varicella, and 10% for flu (Table 2). Overall vaccination confidence was negatively associated with refusal of any vaccine such that every one point increase in the scale mean corresponded with a 42% decrease in the odds of refusal (odds ratio [OR] = 0.58, 95% confidence interval [CI], 0.54–0.63). Vaccination confidence was also negatively associated with refusal of MMR, varicella, and flu vaccines.

**Table 2:** Parent-reported vaccine refusal: Multivariable associations with scale and factor score means (*n* = 9,354).

		Scale		Factors					
	Refused	Confidence (8 items)		Benefits (4 items)		Harms (2 items)		Trust (2 items)	
	n		OR		OR		OR		OR
	(%)	Mean (SE)	(95% CI)	Mean (SE)	(95% CI)	Mean (SE)	(95% CI)	Mean (SE)	(95% CI)
Any vaccine									
Yes	1,429		**0.58**		**0.73**		**1.19**		1.03
	(15%)	7.27 (0.09)	**(0.54, 0.63)**	7.70 (0.10)	**(0.68, 0.78)**	5.28 (0.13)	**(1.15, 1.24)**	8.95 (0.09)	(0.95, 1.11)
No	7,925								
	(85%)	8.48 (0.02)	Reference	8.90 (0.03)	Reference	3.28 (0.05)	Reference	9.41 (0.03)	Reference
MMR									
Yes	261		**0.39**		**0.51**		**1.42**		**1.21**
	(3%)	5.38 (0.23)	**(0.34, 0.45)**	5.38 (0.29)	**(0.45, 0.58)**	7.58 (0.26)	**(1.24, 1.61)**	8.33 (0.22)	**(1.06, 1.39)**
No	9,093								
	(97%)	8.38 (0.02)	Reference	8.81 (0.03)	Reference	3.47 (0.05)	Reference	9.37 (0.03)	Reference
Varicella			s						
Yes	342		**0.42**		**0.53**		**1.41**		**1.25**
	(3%)	5.74 (0.19)	**(0.36, 0.47)**	5.85 (0.24)	**(0.47, 0.59)**	7.32 (0.23)	**(1.27, 1.56)**	8.56 (0.16)	**(1.09, 1.42)**
No	9,012								
	(97%)	8.39 (0.02)	Reference	8.82 (0.03)	Reference	3.45 (0.05)	Reference	9.36 (0.03)	Reference
Flu									
Yes	954		**0.57**		**0.72**		**1.23**		1.06
	(10%)	7.05 (0.10)	**(0.52, 0.62)**	7.47 (0.12)	**(0.67, 0.78)**	5.67 (0.16)	**(1.18, 1.29)**	8.91 (0.09)	(0.96, 1.16)
No	8,400								
	(90%)	8.44 (0.03)	Reference	8.86 (0.03)	Reference	3.34 (0.05)	Reference	9.39 (0.03)	Reference

*Note*: CI: confidence interval. MMR: measles, mumps, and rubella. OR: odds ratio. SE: standard error. Table shows raw frequencies and weighted estimates. Models controlled for child’s race/ethnicity, mother’s educational attainment, and annual household income. Bolded odds ratios indicate statistically significant associations.

In models that included the three factors separately, the Benefits factor was most strongly associated with refusal of any vaccine (OR = 0.73, 95% CI, 0.68–0.78), as well as with the refusal of MMR, varicella, and flu vaccines specifically. The Harms factor was less strongly associated with refusal of any vaccine (OR = 1.19, 95% CI, 1.15–1.24), as well as with the refusal of MMR, varicella, and flu vaccines. Trust was not associated with refusal of any vaccine, but was positively associated with refusal of MMR and varicella vaccines, contrary to our expectations.

### Vaccine delay

Twenty-seven percent of parents reported having delayed any vaccine for their child, with delay of specific vaccines being 8% for MMR, 7% for varicella, and 11% for flu (Table 3). In models using the full 8-item scale, vaccination confidence was negatively associated with delay of any vaccine (OR = 0.81, 95% CI, 0.76–0.86), as well as with delay of MMR, varicella, and flu vaccines.

**Table 3:** Parent-reported vaccine delay: Multivariable associations with scale and factor score means (*n* = 9,354).

		Scale		Factors					
	Delayed	Confidence (8 items)		Benefits (4 items)		Harms (2 items)		Trust (2 items)	
	n		OR		OR		OR		OR
	(%)	Mean (SE)	(95% CI)	Mean (SE)	(95% CI)	Mean (SE)	(95% CI)	Mean (SE)	(95% CI)
Any vaccine									
Yes	2,289		**0.81**		**0.93**		**1.08**		0.96
	(27%)	7.98 (0.06)	**(0.76, 0.86)**	8.45 (0.06)	**(0.87, 0.99)**	4.18 (0.11)	**(1.05, 1.12)**	9.19 (0.06)	(0.89, 1.04)
No	7,065								
	(73%)	8.41 (0.03)	Reference	8.82 (0.03)	Reference	3.37 (0.06)	Reference	9.39 (0.03)	Reference
MMR									
Yes	789		**0.68**		**0.80**		**1.11**		0.98
	(8%)	7.44 (0.13)	**(0.62, 0.74)**	7.84 (0.15)	**(0.73, 0.86)**	4.86 (0.21)	**(1.05, 1.18)**	8.94 (0.16)	(0.86, 1.11)
No	8,565								
	(92%)	8.37 (0.03)	Reference	8.79 (0.03)	Reference	3.47 (0.05)	Reference	9.37 (0.03)	Reference
Varicella									
Yes	619		**0.67**		**0.76**		**1.12**		1.05
	(7%)	7.34 (0.13)	**(0.62, 0.73)**	7.70 (0.15)	**(0.70, 0.82)**	5.04 (0.22)	**(1.06, 1.18)**	9.01 (0.11)	(0.94, 1.18)
No	8,735								
	(93%)	8.37 (0.03)	Reference	8.79 (0.03)	Reference	3.48 (0.05)	Reference	9.36 (0.03)	Reference
Flu									
Yes	997		**0.75**		**0.86**		**1.08**		0.95
	(11%)	7.71 (0.10)	**(0.70, 0.81)**	8.15 (0.11)	**(0.80, 0.93)**	4.47 (0.16)	**(1.03, 1.13)**	9.02 (0.10)	(0.86, 1.04)
No	8,357								
	(89%)	8.37 (0.03)	Reference	8.79 (0.03)	Reference	3.47 (0.05)	Reference	9.38 (0.03)	Reference

*Note*: CI: confidence interval. MMR: measles, mumps, and rubella. OR: odds ratio. SE: standard error. Table shows raw frequencies and weighted estimates. Models controlled for child’s race/ethnicity, mother’s educational attainment, and annual household income. Bolded odds ratios indicate statistically significant associations.

In models using the three factors, Benefits again demonstrated the strongest association with delay of any vaccine (OR = 0.83, 95% CI, 0.87–0.99), as well as with delay of MMR, varicella, and flu vaccines. The Harms factor was positively associated with any vaccine delay (OR = 1.08, 95% CI, 1.05–1.12), as well as with delay of MMR, varicella, and flu vaccines. Trust was not associated with any of the four measures of vaccine delay.

#### Vaccination status

Seventy-four percent of children were up-to-date for the combined vaccine series, with vaccine-specific coverage being 91% for MMR, 91% for varicella, and 35% for flu (Table 4). In models using the full 8-item scale, vaccination confidence was positively associated with coverage for the combined series (OR = 1.35, 95% CI, 1.27–1.44), as well as with coverage of MMR, varicella, and flu vaccines.

**Table 4:** Provider-reported vaccination status: Multivariable associations with scale and factor score means (*n* = 9,354).

		Scale		Factors					
	Vaccinated	Confidence (8 items)		Benefits (4 items)		Harms (2 items)		Trust (2 items)	
	n		OR		OR		OR		OR
	(%)	Mean (SE)	(95% CI)	Mean (SE)	(95% CI)	Mean (SE)	(95% CI)	Mean (SE)	(95% CI)
Combined series^a^									
Yes	7,122		**1.35**		**1.16**		**0.93**		**1.08**
	(74%)	8.47 (0.03)	**(1.27, 1.44)**	8.88 (0.03)	**(1.09, 1.23)**	3.32 (0.06)	**(0.90, 0.96)**	9.43 (0.03)	**(1.01, 1.17)**
No	2,232								
	(26%)	7.85 (0.06)	Reference	8.31 (0.06)	Reference	4.25 (0.11)	Reference	9.05 (0.06)	Reference
MMR									
Yes	8,625		**1.53**		**1.33**		**0.92**		1.04
	(91%)	8.40 (0.02)	**(1.40, 1.68)**	8.83 (0.03)	**(1.23, 1.44)**	3.45 (0.05)	**(0.86, 0.98)**	9.38 (0.03)	(0.94, 1.14)
No	729								
	(9%)	7.39 (0.14)	Reference	7.75 (0.13)	Reference	4.79 (0.25)	Reference	8.84 (0.12)	Reference
Varicella									
Yes	8,512		**1.54**		**1.29**		**0.89**		1.02
	(91%)	8.40 (0.02)	**(1.42, 1.66)**	8.83 (0.03)	**(1.19, 1.39)**	3.42 (0.05)	**(0.84, 0.93)**	9.37 (0.03)	(0.93, 1.12)
No	842								
	(9%)	7.39 (0.11)	Reference	7.81 (0.12)	Reference	4.97 (0.18)	Reference	8.90 (0.09)	Reference
Flu									
Yes	3,400		**1.32**		**1.13**		**0.92**		1.06
	(35%)	8.56 (0.04)	**(1.23, 1.42)**	8.97 (0.04)	**(1.05, 1.22)**	3.18 (0.07)	**(0.89, 0.96)**	9.48 (0.04)	(0.96, 1.17)
No	2,756								
	(33%)	8.03 (0.05)	Reference	8.50 (0.05)	Reference	4.04 (0.10)	Reference	9.17 (0.06)	Reference
Not eligible^b^	3,198								
	(32%)	8.32 (0.04)		8.72 (0.04)		3.50 (0.09)		9.33 (0.05)	

*Note*: CI: confidence interval. MMR: measles, mumps, and rubella. OR: odds ratio. SE: standard error. Table shows raw frequencies and weighted estimates. Models controlled for child’s race/ethnicity, mother’s educational attainment, and annual household income. Bolded odds ratios indicate statistically significant associations.

^a^ Combined series (4:3:1:-:3:1:4) included ≥4 doses of DTaP/DT/DTP, ≥3 doses of poliovirus vaccine, ≥1 dose of measles-containing vaccine, ≥3 doses of HepB, ≥1 dose of varicella vaccine, and ≥4 doses of PCV. Hib excluded due to shortage.

^b^ Not eligible group excluded from logistic regression models. This group consisted of children who were not 6- to 23- months of age for September 1 through December 31 in the year prior to data collection.

In models using the three factors, Benefits was positively associated with coverage for the combined series (OR = 1.16, 95% CI, 1.09–1.23), as well as coverage of MMR, varicella, and flu vaccines. The Harms factor was negatively associated with coverage for the combined series (OR = 0.93, 95% CI, 0.90, 0.96), as well as coverage of MMR, varicella, and flu vaccines. Trust was associated with coverage for the combined series only (OR = 1.08, 95% CI, 1.01–1.17).

## Discussion

Using data from a large, nationally-representative sample of parents, we found that vaccination confidence was consistently associated with behaviors related to early childhood vaccination. Mean scores on the Vaccination Confidence Scale were most strongly associated with measures of vaccine refusal, with each one point increase in mean scale scores corresponding with a reduction in the odds of refusal ranging from 42% for any vaccine to 61% for MMR. Although we identified a similar pattern of findings in a prior study focusing on adolescent vaccines [9], the associations between mean scale scores and measures of vaccine refusal were greater in magnitude for the early childhood vaccines that were the focus of the present study. These findings suggest that vaccination confidence may be particularly salient for parents of young children, who are faced with relatively frequent decisions about whether or not to accept vaccinations [13–17].

In addition to vaccine refusal, we found that vaccination confidence was consistently associated with measures of vaccine delay and vaccination status. Each one point increase in mean scale scores was associated with a reduction in the odds of delay ranging from 19% for any vaccine to 33% for varicella vaccine. Here again, our scale appeared to perform more strongly for early childhood vaccines than adolescent vaccines, as our prior study found that scale scores were only weakly and inconsistently associated with adolescent vaccine delay [9]. In the case of vaccination status, each one point increase in mean scale scores was associated with an improvement in the odds of vaccination ranging from 32% for flu vaccine to 54% for varicella vaccine. These findings on early childhood vaccination status are comparable to the associations we observed in our prior study of adolescent vaccines [9].

When we examined the performance of individual factors within our scale, we found that, across our measures of refusal, delay, and vaccination status, the Benefits factor was most strongly associated with vaccination behavior. As in our prior study [9], our findings suggest that the Benefits items offer a possible “short form” of our scale. The Harms factor was also associated with these measures, although somewhat less strongly. These findings are consistent with the well-established literature on the Health Belief Model, which documents the influence of perceived risks and benefits on vaccination behavior [13,18–19]. In contrast to Benefits and Harms, we were surprised to note that the Trust factor was associated with only three measures of vaccination behavior, and in two instances, these associations were not in the expected direction. Given the extent to which prior research has emphasized the importance of the parent-provider relationship in parents’ vaccine-related decision making [13,20–22], our findings likely reflect shortcomings in our measure of trust. For example, our measure was specific to healthcare providers; trust in other entities, such as pharmaceutical companies or governmental bodies, may also be relevant, as well as trust in vaccines themselves. Furthermore, the high mean value for this construct suggests a possible ceiling effect, whereby respondents endorsed trust items so highly that meaningful variation was lacking. Future research can extend the present study by developing and evaluating items that better capture variation with regard to parents’ trust of their children’s vaccine providers or the healthcare system more generally.

Overall, our findings provide strong support for broadening the application of the Vaccination Confidence Scale to measure parents’ beliefs about early childhood vaccination. Specifically, we anticipate that our brief scale can complement existing measures [23–26] to expand our ability to conduct surveillance of vaccine hesitancy, a goal prioritized by the NVAC Vaccine Confidence Working Group [7]. Similarly, our scale could also be used for evaluating interventions to address vaccine hesitancy and for segmenting audiences to facilitate the development and delivery of messages targeted by parents’ level of vaccination confidence. Such targeting is important because parents’ informational needs vary according to the extent of their vaccine-related concerns [27]. Although the Vaccination Confidence Scale may have clinical applications as a screening tool to identify parents at risk for refusing or delaying early childhood vaccines, additional research will be needed to assess the effectiveness of using our scale as an individual- versus population-level measure.

Strengths of this study include the validation of our scale with regard to vaccine refusal and delay, two behaviors that are more relevant than vaccination status alone to understanding parents’ participation in immunization programs. This study is also strengthened by the use of data from the NIS, a large, nationally-representative survey of parents which also offers provider-reported data on children’s vaccination status. Limitations of this study include its cross-sectional design, which limits our ability to establish the directionality of the relationship between vaccination confidence and behavior. Future research can build upon the present study by prospectively assessing Vaccination Confidence Scale scores and subsequent refusal or delay of early childhood vaccines. This study was also limited by its focus on parents’ vaccination beliefs. Although this focus served the study’s primary goal of validating the Vaccination Confidence Scale, we acknowledge that other factors, such as provider recommendations and clinical systems for patient recall and reminders, are also important determinants of early childhood vaccination coverage [28–31].

In conclusion, the findings of this study suggest that vaccination confidence is closely tied to early childhood vaccination behavior. Parents’ mean scores on the 8-item Vaccination Confidence Scale were consistently associated with vaccine refusal, vaccine delay, and vaccination status across a variety of early childhood vaccines. Indeed, although we originally developed our scale to investigate parental beliefs about adolescent vaccines, the findings of this study suggest that our measure is even more relevant to the context of early childhood vaccination. Additional prospective research will be needed to characterize causal pathways between vaccination confidence and behavior, but we believe that the present study suggests considerable promise for using the Vaccination Confidence Scale as a brief measure of vaccination beliefs relevant to vaccine hesitancy. In this way, our scale can offer a measurement tool to support public health efforts to build parents’ confidence in immunization programs so as to protect individuals and communities from vaccine preventable diseases.
